# Occurrence, Sources, and Risk Assessment of PFAS in Soil–Mango Systems of the Chinese Tropical Nanfan District

**DOI:** 10.3390/foods15010058

**Published:** 2025-12-24

**Authors:** Zhen Zhang, Fei Chen, Rui Yang, Saihao Ren, Shanying Zhang, Xiaowei Pan, Hai Tian, Thiagarajah Ramilan, Yun Duan, Bingjun Han

**Affiliations:** 1 Analysis and Test Center, Chinese Academy of Tropical Agricultural Sciences, Key Laboratory of Quality and Safety Control for Subtropical Fruit and Vegetable, Ministry of Agriculture and Rural Affairs, Hainan Provincial Key Laboratory of Quality and Safety for Tropical Fruits and Vegetables, Key Laboratory of Nutritional Quality and Health Benefits of Tropical Agricultural Products of Haikou City, Laboratory of Quality & Safety Risk Assessment for Tropical Products (Haikou), Ministry of Agriculture and Rural Affairs, Haikou 571101, China; 18145123688@163.com (Z.Z.); tianhai666@163.com (H.T.); 2 School of Food Science and Engineering, Hainan University, Haikou 570228, China; 18299151539@163.com; 3 Hainan Ecological Environmental Monitoring Center, Haikou 572000, China; 21180311019@stu.ouc.edu.cn; 4 Agricultural Products Processing Research Institute, Chinese Academy of Tropical Agricultural Sciences, Zhanjiang 524001, China; rshkyzy@foxmail.com (S.R.); panxiaowei@catas.cn (X.P.); 5 Sanya Nanfan Research Institute of Hainan University, School of Tropical Agriculture and Forestry (School of Agricultural and Rural Affairs, School of Rural Revitalization), Hainan University, Haikou 570228, China; 17889986721@163.com; 6 School of Agriculture and Environment, Massey University, Palmerston North 4442, New Zealand; t.ramilan@massey.ac.nz

**Keywords:** perfluoroalkyl and polyfluoroalkyl substance, soils, mango, bioaccumulation factor, risk assessment, pollution sources

## Abstract

Perfluoroalkyl and polyfluoroalkyl substances (PFASs) have emerged as contaminants of global concern due to their persistence and potential health risks. PFASs pose potential pollution risks in mango cultivation and production. This study investigated pollution characteristics and conducted a comprehensive risk assessment of PFASs in soil–mango systems within the Nanfan District of Hainan, China. The results revealed that total PFAS concentrations in soil ranged from 0.18 to 1.07 ng/g, with PFHpA and PFHxA accounting for 24.9% and 21.0%, respectively. Total PFAS concentrations in mangoes ranged from 0.0019 to 0.0201 ng/g wet weight, where PFHxA and PFHpA accounted for 44.02% and 30.28%, respectively. For all PFASs, the bioaccumulation factor (BAF) in mangoes was <1, indicating limited transfer from soil to fruits. Regarding PFAS contamination sources, long-range atmospheric transport may serve as the primary pathway for PFAS contamination in soil and mangoes. Risk assessments indicated minimal ecological and dietary exposure risks, with soil ecological risk quotients (RQs) below 0.01 and edible exposure RQs below 1. This study highlights the unique contribution of short-chain PFAS to the quality and safety of tropical agricultural products and provides critical data for the safety regulation of PFASs in soil–fruit systems.

## 1. Introduction

The contamination of perfluoroalkyl and polyfluoroalkyl substances (PFASs) in global agricultural production has become a significant challenge, threatening the quality and safety of agricultural products. As a core region of China’s tropical agriculture, Hainan Nanfan District hosts a pivotal mango industry that not only serves as the backbone of the regional economy but also directly impacts the health of domestic and foreign consumers through its product quality and safety. It is worth noting that mango bags are extensively used during planting. These bags function as physical protection, pest control tools, and quality-enhancing measures for fruits. However, their widespread application may introduce PFAS contamination in mangoes. In the tropical field environment, the PFAS commonly present in the waterproof and oil-resistant coatings of bag materials may migrate into mangoes under the influence of temperature (25–35 °C) and humidity (60–85%) [1], thereby posing potential health risks to consumers. Therefore, the unique climate and agricultural management model in Nanfan District provides a typical scenario for studying the occurrence and migration of PFAS in tropical soil–mango systems.

In recent years, environmental pollution and potential health risks induced by PFAS have emerged as a global concern. As a class of synthetic pollutants with high persistence, PFASs are widely distributed in various food matrices, including fruits, vegetables, fish, and dairy products. Dietary intake constitutes the primary route of human exposure, and the association between their environmental migration and food contamination has rendered related research a critical topic in the field of food safety [2,3]. The bioaccumulative properties of PFAS are closely linked to developmental toxicity, immunotoxicity, and hepatotoxicity in both animals and humans. Relevant studies have demonstrated that PFASs can bind to proteins in the human body, predominantly accumulating in tissues such as blood, liver, and kidneys, with extremely low metabolic excretion efficiency. Long-term accumulation may amplify their toxic effects [4]. A growing body of evidence confirms that PFAS exposure exerts significant multisystem toxicity: In the metabolic system, it can induce dyslipidemia (e.g., hyperlipidemia), insulin resistance, and gestational diabetes. Furthermore, it may interfere with mitochondrial DNA (mtDNA)-mediated regulatory pathways, thereby altering the metabolic levels of triglycerides (TGs), total cholesterol (TC), and low-density lipoproteins (LDLs) in Chinese children aged 7–10 years [5]. In the endocrine and immune systems, it can cause thyroid dysfunction, immunosuppression, and chronic low-grade inflammation. It may even modulate the TNF and IL-17 signaling pathways, affecting the systemic immune-inflammation index (SII) and triglyceride–glucose index (TyG), which in turn increases the risk of kidney stones [6]. In reproductive and digestive systems, it may further elevate the risk of liver damage, colitis, and preeclampsia, while long-term exposure is more likely to increase the incidence of metabolism-related diseases and cancer [2,3,7]. Notably, vulnerable populations, including pregnant women, neonates, and children, exhibit high susceptibility to PFASs. Early-life exposure may impose irreversible long-term impacts on growth and development. Given the combined hazards of PFAS to multiple human systems and their widespread occurrence in the food chain, conducting research on PFAS pollution source apportionment, health risk assessment, and prevention and control technologies has become an urgent priority in the fields of environmental science, public health, and food safety.

Soil is an important reservoir of PFAS in terrestrial ecosystems, and the PFASs contained in it constitute a significant source of PFAS contamination for plants. PFASs in soil can be absorbed by plant roots and transported to various parts of the plant. The absorption mechanism is complex, involving passive diffusion and active transport. Factors such as growth stage, temperature, humidity, and soil characteristics all influence the transfer of PFASs from soil to plants [8]. Different plant species have varying accumulation levels, with some plants being more prone to accumulation than others. In soil severely contaminated with PFASs, long-chain PFAS tend to accumulate in the roots of cereal crops and vegetables, while short-chain homologues are more likely to transfer to the above-ground parts [9]. In such soil, the content of perfluoro-n-octanoic acid (PFOA) in wheat can reach up to 580 µg/kg, and the total concentration of PFAS can be as high as 2597 µg/kg [10]. However, these studies only focused on crops from contaminated areas and cannot reveal the actual accumulation levels of PFASs in the agricultural products we consume daily. Moreover, research on PFAS contamination in tropical fruits is limited, and it remains unclear whether the accumulation trend in PFASs in mangoes matches that in other plants or how soil pH and organic matter affect the transfer of soil-derived PFASs to mangoes. Given the possibility of PFAS accumulation in plant tissues and the high dietary intake of mangoes, understanding the presence and concentration of PFASs in mangoes is crucial for accurately assessing potential health risks to humans. Therefore, it is necessary and urgent to investigate and identify typical PFAS in mangoes from normal farmlands.

In this study, the pollution characteristics and potential risks of PFASs in soil and mango were investigated in Nanfan District in Hainan, China. The purpose of this study was to (i) explore the main species, content level, and enrichment characteristics of PFASs in soil and mango; (ii) explore the effects of soil physicochemical properties on the accumulation of PFASs; (iii) investigate the sources of PFAS contamination in soil and mango; and (iv) assess the ecological and human health risks of PFASs. This will clarify the environmental fate and risk level of PFASs in the soil–mango system. This study offers basic data and technical support for the safety supervision of tropical agricultural products and the management and safe utilization of PFAS-polluted soil. It holds significant importance for the development of the discipline of agricultural environmental protection and ensuring the quality and safety of agricultural products.

## 2. Materials and Methods

### 2.1. Chemicals and Reagents

In this study, 8 target PFAS compounds and isotope labeling standards (>98%) listed in Table S1 were purchased from Alta Scientific Co., Ltd. (Tianjin, China). The mixed target perfluoroalkyl analytes (at 50 ng/mL), surrogate mass-labelled compounds (at 50 ng/mL), and injection standards (at 100 ng/mL) were prepared in methanol. Methanol and acetonitrile (both LC-MS grades) were supplied by CNW Technologies (Shanghai, China), while formic acid (≥99%) and acetic acid (≥99%) were supplied by RHAWN (Shanghai, China). Ammonia (LC-MS grades) was supplied by Aladdin (Shanghai, China). Mobile phase water is produced by Arium ultrapure Water Systems (Sartorius, Goettingen, Germany). Activated carbon powder was obtained from Merck (Darmstadt, Germany). Ammonium acetate was supplied by Sigma-Aldrich (St. Louis, MO, USA). Weak anion exchange solid-phase extraction (SPE) cartridges (150 mg, 6 mL) were manufactured by Agilent Technologies (Santa Clara, CA, USA). 

### 2.2. Sample Collection

This sampling campaign aimed to systematically evaluate the fruit and soil conditions in major mango-producing regions of the Nanfan District of Hainan, China. The selection of sampling sites adhered to the principles of representativeness, comprehensiveness, and feasibility: Core planting areas were selected across four counties (Lingshui, Sanya, Ledong, and Dongfang) to ensure coverage of typical local production models, soil types, and cultivated varieties, thereby reflecting the overall mango production status of each administrative region. Within each county, sampling sites were evenly distributed across major orchards in different towns and townships, achieving reasonable spatial and ecological coverage. A total of 24 mango fruit samples and 26 soil samples were collected. Among these, sampling sites 1–24 yielded paired fruit and rhizosphere soil samples for direct correlation analysis; sites 25–26 were supplementary soil-only sampling points to enhance the representativeness of regional soil background data. The sample size was determined based on research objectives and statistical requirements: The 24 paired samples provided a foundation for correlational analysis between soil and fruit parameters, enabling the identification of key association patterns in this exploratory study. By covering diverse major planting areas across the four regions—encompassing critical sources of variation such as geographical distribution, cultivar types, and management practices—the samples effectively reflect regional-scale characteristics. Given this study’s focus on revealing underlying patterns and significant differences rather than fine-scale regional mapping, the current sample size, while ensuring operational feasibility, is sufficient to support inter-city/county statistical comparisons and multivariate analyses (e.g., principal component analysis), thereby guaranteeing the scientific reliability of the results.

Sampling was conducted in May 2024, during the peak ripening period of mangoes in Hainan. At this stage, fruit quality traits are stable, facilitating the collection of samples that accurately reflect actual production levels. Concurrently, the soil environment is in a relatively stable state during the mango growing season, which is conducive to assessing the current status of orchard soils and their potential relationships with fruit quality. This ensures the typicality of the samples and the timeliness of the data. At the time of harvest, the mangoes exhibited purplish-red peels, pale yellow flesh, rounded fruit shoulders, and partial bright red coloration in some areas. Mango bags from 26 sampling sites were identical, manufactured by Jintian Mango Packaging Factory (Sanya, China) using raw wood pulp as the main material. In this study, mango bag samples D1, D2, and D3 were collected from site 12, site 15, and site 18, respectively. The 0–20 cm topsoil was collected with a stainless-steel shovel, and mango samples were stored in polypropylene (PP) zip-lock bags alongside the soil samples. The researchers avoided materials containing PFASs during the sampling process. All samples collected were kept in an ice box during transport and then stored in the -20 °C lab refrigerator until analysis. Soil pH and soil organic matter were analyzed, with analytical methods detailed in the Supplementary Materials.

### 2.3. Preparation of Soil and Mango Bag Samples

The collected soil samples were freeze-dried, homogenized in an agate mortar, and sifted through a 149 μm screen. The 5.0 g soil sample was accurately weighed and transferred into a 50 mL polypropylene tube. Then, 500 μL of 2 ng/L isotopically labeled standard solution (substitute standard) and 10 mL of methanol aqueous solution (methanol, 50 vol%) were added for vortex mixing. The mixture was oscillated horizontally for 2 h and centrifuged at 9000 rpm for 10 min. The supernatant was transferred to another polypropylene tube. Then, 10 mL of 50 vol% aqueous methanol solution was added to the tube containing the residual sample for repeated extraction. The extracts were combined twice. The extraction solution was filtered and diluted with 60 mL of water. Prior to purification, SPE cartridges were activated in sequence with 6 mL of 2 vol% NH_3_·H_2_O–methanol solution, 6 mL of methanol, and 6 mL of distilled water. After the diluted extract was passed through an SPE cartridge, the cartridge was washed with 6 mL of distilled water and 8 mL of ammonium acetate buffer, dried under vacuum for 10 min, washed with 8 mL of methanol, and finally eluted with 6 mL of ammonia–methanol solution to collect the eluent. The eluent was dried under a gentle nitrogen stream, redissolved in 1 mL of methanol, mixed thoroughly, and stored in a sample vial. Mango bags were cut into small pieces, and 1 g of samples was extracted using the same procedure described above.

### 2.4. Mango Sample Preparation

The extraction method for PFAS from mangoes was slightly modified from a previous study [11]. Briefly, all edible portions of the mango samples were homogenized. Then, a 5 ± 0.10 g portion of mango homogenized tissue (wet weight, ww) was accurately weighed and transferred into a 50 mL polypropylene tube. Then, 500 μL of a 2 ng/L isotopically labeled standard solution (substitute standard), 10 mL of 1.5 vol% formic acid in acetonitrile, 5 mL of distilled water, and 100 mg of activated carbon powder were added. The mixture was horizontally shaken for 40 min and centrifuged at 8000 rpm for 15 min, after which the supernatant was collected for purification. Prior to purification, an SPE cartridge was activated in sequence with 5 mL of methanol and 3 mL of 2% aqueous formic acid. The supernatant was passed through the SPE cartridge, followed by washing with 3 mL of 2% aqueous formic acid and 3 mL of methanol. The cartridge was dried for 15 min and eluted twice with 2 mL of 10 vol% NH_3_·H_2_O–methanol solution. The eluate was dried under a nitrogen stream at 45 °C and redissolved in 300 μL of 10 vol% aqueous methanol for analysis.

### 2.5. UPLC-MS/MS Experimental Condition

The analysis of PFASs was performed on an SCIEX Triple Quad 6500+ UPLC-triple quadrupole tandem mass spectrometry system. The separation of target PFAS was performed on a ZORBAX Eclipse Plus C18 column (2.1 mm × 100 mm, 1.8 µm, Agilent Technologies, USA). The ZORBAX Eclipse Plus C18 column (2.1 mm × 50 mm, 1.8 µm, Agilent Technologies, USA) served as the trap column to capture any system-related disturbances. The mobile phase consisted of methanol (mobile phase A) and 2 mM ammonium acetate solution (mobile phase B). The column temperature was set at 35 °C; the injection volume was 5.0 μL; the flow rate was 0.3 mL/min. The gradient elution program was as follows: 30–60% A from 0 to 7 min, 60–95% A from 7 to 13 min, 95% A from 13 to 16 min, 95–30% A from 16 to 16.1 min, and 30%A from 16.1 to 20 min. The mass spectrometer was operated in negative electrospray ionization (ESI-) mode with the following parameters: multiple reaction monitoring (MRM); capillary voltage, 2500 V; vacuum interface temperature, 200 °C; desolvation temperature, 350 °C; nebulizer gas flow, 1.0 L/min; desolvation gas flow, 15 L/min; backblow gas flow, 1.5 L/min; collision gas flow, 0.25 mL/min.

### 2.6. Quality Assurance and Quality Control

The containers used during sample preparation and analysis were washed with HPLC-grade methanol and Milli-Q water, and operators avoided using materials containing polytetrafluoroethylene (PTFE) and PFAS-related experimental materials to prevent background contamination. To check for carryover and background contamination during instrument analysis, solvent blanks (mass spec grade methanol) were run on 15 samples per batch. The matrix-dependent method detection limits were 0.0648 × 10^−3^–1.02 × 10^−3^ ng/g ww, and blank test results were below the method detection limits. The limit of quantitation (LOQ) and limit of detection (LOD) were determined with signal-to-noise ratios of 10:1 and 3:1, respectively. Eight calibration curve points were prepared using the internal standard method (0.05, 0.125, 0.25, 0.5, 1, 2.5, 5, and 10 ng/mL), and the correlation coefficient (r2) of each calibration curve was greater than 0.99. The recovery rate of spiked samples at different concentrations (0.5, 10, 25, and 50 ng/g) ranges from 82.9% to 113.0%. Detailed QA/QC information can be found in Table S2.

### 2.7. Bioaccumulation Factors of PFAS

The bioaccumulation factor (BAF) is expressed as the ratio of target PFAS concentration in the mango to target PFAS concentration in the corresponding soil. BAF was calculated using the equation as follows [12]:

$$BAF=\frac{PFAS\;concentration\;in\;mango\;\left(\frac{ng}{g}ww\right)}{PFAS\;concentration\;in\;mango\;soil\;\left(\frac{ng}{g}dw\right)}$$

BAF > 1 indicates that the content of the compound in mango is greater than that in soil, which indicates that the compound is more easily transported and accumulated in mango.

### 2.8. Ecological Risk Assessment of Soil PFAS

The potential ecological risk of soil PFASs was assessed by calculating risk quotient (RQ) values:

$$RQ=\frac{MEC}{PNEC}$$

MEC is the actual measured concentration, and PNEC is a predicted no-effect concentration. When 0.01 < RQ < 0.1, the risk is low. When 0.1 < RQ < 1, it is medium risk. When RQ > 1, it is high risk [13].

### 2.9. Dietary Risk Assessment of PFASs

Mango consumption is considered to be one of the key pathways for human exposure to PFASs. When calculating the total dietary intake of PFAS in mangoes, this study divided the population into three age groups and took the average age of each age group as the core calculation parameter so as to obtain the following equation of the estimated daily intake (EDI) of PFAS affected by weight factors for the three age groups:

$$EDI=\frac{DC\times C}{BW}$$

where C represents the average concentration of individual PFAS in mangoes (ng/g); DC is the average daily consumption rate of mangoes in the target population, which is 0.6899 g/kg·bw/d; BW is the average body weight of the target consumer population, with the mean body weight of males being 69.6 kg and that of females being 59 kg. EDI estimation parameters were derived from the China Health and Nutrition Survey (CHNS) and the China Food Composition Table (CFCT).

The RQ is calculated from the following formula:

$$RQ=\frac{EDI}{RfD}$$

Because the United States Environmental Protection Agency (USEPA) has only developed an oral RfD reference dose for some PFASs, the only RfD values for PFOA, PFOS, and PFHxA were 3 ng/kg/day, 2 ng/kg/day [14], and 320 ng/kg/day [12], respectively, for this study. An RQ value greater than 1 indicates a higher risk of exposure to PFAS via ingestion, while an RQ value less than 1 indicates a lower risk.

### 2.10. Data Analysis

In this study, IBM SPSS statistics 27.0, Origin 2021, and Excel 2021 were used for statistical testing, plotting, and data processing. Significant differences were identified based on significance levels (*p* < 0.01, *p* < 0.05). Principal component analysis (PCA) was performed using SPSS 27.0 software to investigate the potential sources of PFAS in soil. Initially, a logarithmic transformation was applied to the raw PFAS concentration data to mitigate the effects of strong right-skewness and extreme value interference. Subsequently, the Kaiser–Meyer–Olkin (KMO) measure of sampling adequacy and Bartlett’s test of sphericity were conducted on the transformed PFAS variables to verify the dataset’s suitability for factor analysis. The factor extraction method was set to “principal components”, with eigenvalues greater than 1 adopted as the criterion for determining the number of factors. Finally, “no rotation” was selected to retain the original orthogonal structure of the extracted factors, and the factor loading matrix was generated for the source apportionment of soil PFASs.

## 3. Results and Discussion

### 3.1. Analysis of Pollution Characteristics of PFASs in Soil

**Figure 1:**
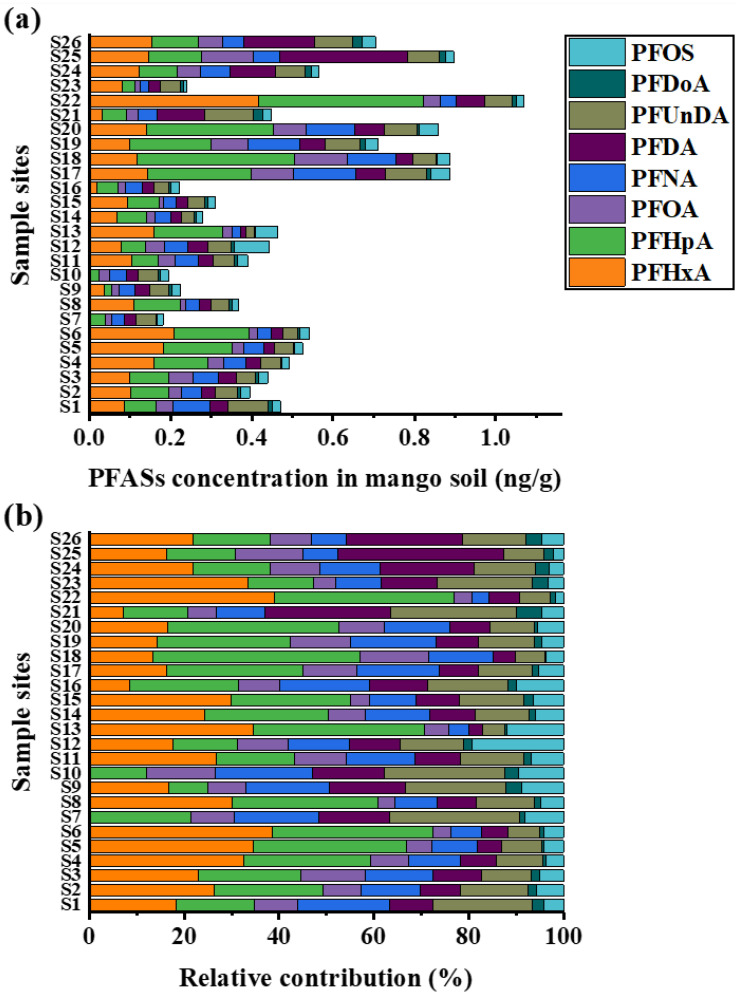
Concentration and proportion of PFAS components in soil. (**a**) The sum of all PFAS concentrations; (**b**) proportion of PFASs.

Targeted PFASs in soil are divided into six legacy PFAS (PFOA, PFNA, PFDA, PFUnDA, PFDoA, and PFOS) and two short-chain PFASs (PFHxA and PFHpA). As shown in Figure 1a, the total concentration of PFASs (ΣPFAS) in 26 soil samples ranged from 0.18 to 1.07 ng/g, with a median value of 0.46 ng/g. The detection rate of PFAS in soil ranged from 92% to 100%, and the average concentration ranged from 0.008 to 0.123 ng/g. As illustrated in Figure 1b, within the composition of PFAS, PFHpA exhibited the highest proportion, accounting for 24.9% of the total PFAS, followed by PFHxA at 21.0%. The other three PFAS congeners with relatively high abundances were PFDA (12.48%), PFNA (12.46%), and PFUnDA (12.37%). In legacy PFAS, PFNA (0.020–0.153 ng/g), PFDA (0.014–0.314 ng/g), and PFUnDA (0.021–0.119 ng/g) were the main compounds, and the average concentrations were 0.060, 0.061, and 0.060 ng/g, respectively. The median concentrations were 0.051, 0.037, and 0.052 ng/g, respectively. In legacy PFAS, PFUnDA was positively correlated with PFNA, PFDA, and PFDoA (*p* < 0.01), as shown in Table S3. All of them were long-chain perfluoroalkyl carboxylic acids with similar carbon chain length and perfluoroalkyl chain structure. Thus, this homology indicates that they have similar environmental behaviors [15]. PFHxA (not detected–0.418 ng/g) and PFHpA (0.019–0.405 ng/g) were the short-chain PFAS in soil; the detection rate was ≥92%, and the average concentration was 0.124 and 0.132 ng/g, respectively. The median concentrations were 0.111 and 0.096 ng/g, respectively. As shown in Table S3, PFHxA and PFHpA exhibited a significant positive correlation (*p* < 0.01). Both PFHxA and PFHpA were short-chain perfluoroalkyl carboxylic acid compounds with similar molecular structures, perfluoroalkyl chains, and carboxylic terminals, so they have homology [15]. In this study, the concentration of two short-chain PFAS congeners in soil was approximately twice that of six legacy PFAS congeners. Moreover, with increasing accumulation of these short-chain PFAS in soil, their ecological risks may progressively surpass those posed by the six legacy PFAS.

PFAS contamination in agricultural soils has been a major concern worldwide. Previous studies have shown that the range of total concentrations of PFAS varies widely in soils grown with various crops in different regions. For example, the total concentration of PFAS in agricultural soils in some temperate areas where wheat is grown ranges from 0.34 to 1.59 ng/g [8], the total concentration of PFAS in agricultural soils in Tianjin, China, ranges from 0.21 to 4.16 ng/g [16], and the total concentration of PFAS in agricultural soils in coastal provinces of eastern China ranges from 0.018 to 1.95 ng/g [17]. Among these studies, legacy PFASs such as PFOS and PFOA are the main types, accounting for about 60–70% of the total. Compared with the above normal agricultural soils, the overall concentrations of PFAS in the soil for mango base in Nanfan District seem to be in a similar range. However, the composition feature that the concentrations of short-chain PFASs are inverted compared with those of legacy PFASs is rather significant. Soil PFAS pollution levels in the mango base are relatively low compared to soils heavily contaminated by long-term industrial emissions. Such industrially polluted soils are typically dominated by legacy PFASs [18]. However, the accumulating trend of short-chain PFASs in mango base soils is a cause for concern. The concentration of short-chain PFASs is higher than that of legacy PFASs. This can be attributed to several factors. First of all, due to the well-known environmental risks of traditional PFASs, they are being phased out globally. Meanwhile, short-chain PFASs are increasingly being used as substitutes in various industries and agricultural production processes [19]. During mango cultivation, the use of pesticides and fertilizers containing short-chain PFASs may lead to elevated levels of these short-chain PFASs in the soil [20]. Second, the environmental behavior of short-chain PFASs, such as their higher mobility under tropical climate conditions and stronger potential for interaction with soil components, may prompt their preferential accumulation [21]. As a result, the risks associated with short-chain PFASs are gradually rising. Given their potential unique toxicological profile and environmental fate, they may pose different ecological and human health threats from legacy PFASs. Future research should focus on elucidating these risks and developing targeted mitigation strategies.

### 3.2. Analysis of PFAS Pollution Characteristics in Mango

**Figure 2:**
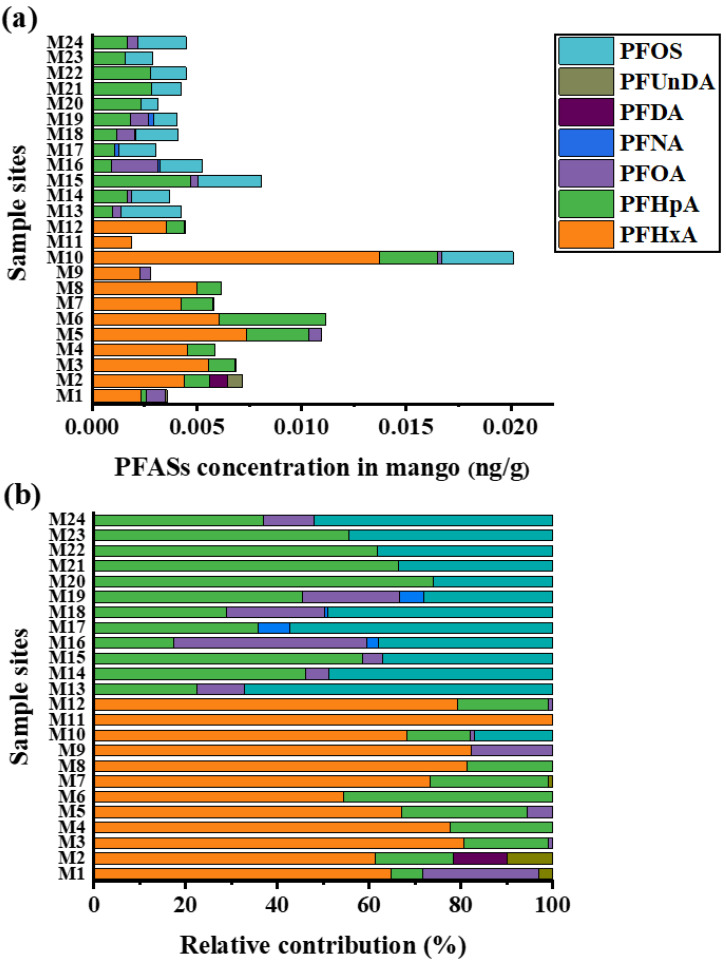
Concentration and proportion of PFAS components in mango. (**a**) The sum of all PFAS concentrations; (**b**) proportion of PFASs.

The analysis results of PFAS in mangoes are reported based on wet weight. PFASs were also widely detected in mangoes, with a detection rate of 100%. As shown in Figure 2a, the total concentration of PFASs in mangoes ranged from 0.0019 to 0.0201 ng/g, with a median value of 0.0045 ng/g. As detailed in PFAS compositions in Figure 2b, PFHxA accounted for the highest proportion, reaching 44.0%, followed by PFHpA at 30.3%. The other two PFAS congeners with relatively high abundances were PFOS (18.43%) and PFOA (5.59%). Mangoes from M1–M12 (Dongfang and Ledong) predominantly contained short-chain PFASs, specifically PFHxA and PFHpA. This compositional profile is consistent with that of PFAS in the corresponding soil samples. In contrast, mangoes from M13 to M24 (Sanya and Lingshui) were characterized by PFHpA and PFOS. This divergence reflects distinct pollution sources. PFOS, a typical long-chain PFAS, is strongly associated with intensive urban activities and higher economic development [22]. As renowned tourist cities in China, Sanya and Lingshui exhibit high population mobility and relatively developed economies, resulting in elevated PFOS inputs. In contrast, Dongfang City and Ledong County have lower industrial and urban emissions, resulting in a lower level of PFOS contamination in M1–M12 mangoes. Although both PFHxA and PFHpA belong to short-chain PFASs, they exhibit significant differences in water solubility. Specifically, as the carbon chain length of PFAS increases, PFAS hydrophobicity enhances while water solubility decreases; therefore, the water solubility of PFHxA is higher than that of PFHpA [23]. The eastern coast of Hainan Island (including Lingshui and Sanya) generally receives more abundant rainfall than the western coast (including Ledong and Dongfang) [24]. The greater rainfall may exert both dilution and leaching effects on PFHxA in mangoes, leading to a significantly lower level of PFHxA contamination in M13–M24 mangoes compared with M1–M12 mangoes. One of the reasons why the PFAS composition characteristics of mangoes in some regions have not shown complete consistency with those of their corresponding soils lies in the differences in their environmental exposure patterns: mangoes are only affected by the environment during the periodic stage from growth to harvest, whereas soils remain under long-term continuous environmental exposure.

The total concentration of short-chain PFASs (PFHxA and PFHpA) was 2.9-fold higher than that of legacy PFAS. The mean concentrations of PFHxA and PFHpA were 0.0051 ng/g and 0.0019 ng/g, respectively. In contrast, PFOA and PFOS exhibited mean concentrations of 0.0006 ng/g and 0.0020 ng/g, respectively. These results indicate that PFHxA and PFHpA exhibited more severe contamination in mangoes. Compared with crops grown in normal agricultural soil, there are some similarities and differences in concentration and composition (Table 1). A study conducted in the Netherlands revealed that fruits and vegetables collected from areas farther away from regions with low background concentrations of PFAS in soil exhibited a mean total PFAS concentration of 0.009 ng/g. Fruits (e.g., strawberries, apples, pears) and leafy vegetables showed higher total PFAS concentrations, yet all values remained below 0.03 ng/g. No distinct dominant PFAS were observed in these fruits [25]. In a parallel investigation using identical analytical methods, food samples from supermarkets, markets, and grocery stores demonstrated low PFAS levels in vegetables, with a mean total concentration of 0.007 ng/g. Legacy PFASs such as PFUnDA, PFOS, and PFDA constituted the major components in vegetables [26]. In comparison with these findings, the PFAS concentrations in mangoes were at comparable levels, yet substantial differences in compositional profiles were observed. The contamination pattern of short-chain PFASs in mangoes was similar to that in crops grown in PFAS-contaminated soil, with both showing relatively high contamination levels (Table 1). Previous studies documented that maize kernels and cabbages near fluorochemical industrial parks contained PFAS concentrations ranging from 1.36 to 58.83 ng/g and 11.88 to 115.14 ng/g, respectively, with short-chain PFASs (PFBA) accounting for higher proportions than legacy PFASs [10,27]. These results indicate widespread production and utilization of emerging substitutes in these regions. In contrast to the aforementioned research findings, there are virtually no fluorine chemical plants located near the mango cultivation areas in Hainan. This implies that the elevated levels of short-chain PFASs in mangoes could be attributed to bioaccumulation and contamination from agricultural inputs.

### 3.3. Bioaccumulation Characteristics of PFASs in Mango

**Figure 3:**
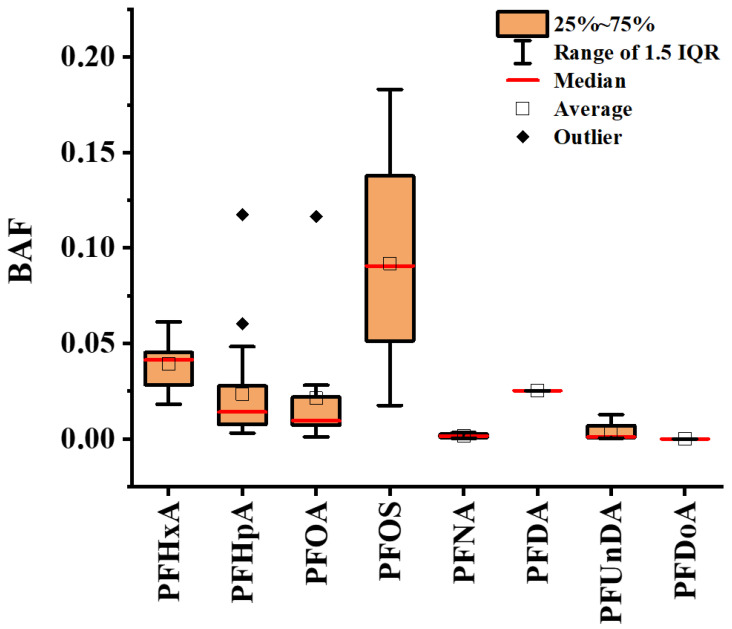
BAF of PFAS in mango. The box and whisker chart displays the BAF values of individual PFAS. The boxes represent the 25th to 75th percentile lines. The solid red line inside the box represents the median, and the black hollow box represents the average value.

The BAF results for PFAS in mangoes are presented in Figure 3. All detected PFASs exhibited BAF values of <1, whereas BAF > 1 indicates facile soil-to-biosystem transfer. These results demonstrate a relatively weak accumulation potential of target PFASs in mangoes. Mango BAF profiles closely mirrored those observed in citrus fruits, where PFASs with varying chain lengths and functional groups showed low bioaccumulation potential [28]. These collective observations suggest thattransport barriers exist during root-to-fruit translocation. Specifically, the endodermis acts as a selective barrier in roots, while complex vascular systems may restrict PFAS movement to fruits. Such barriers likely impede effective transfer, resulting in low accumulation in pulp tissues [10]. Consequently, only minimal PFAS quantities reach reproductive organs. Additionally, the xylem-to-phloem transition represents a critical bottleneck: After xylem transport to leaves, PFAS must enter phloem to reach sinks [29]. In dicotyledonous plants such as mangoes, PFAS must penetrate the cambium (a specialized meristematic tissue forming an additional structural barrier between xylem and phloem) to reach the fruit [30]. This explains why the content of PFASs is low in the fruits of crops such as tomatoes, peas [31], potato tubers [32,33], and mangoes. As shown in Figure 3, from left to right, they are PFHxA, PFHpA, PFOA, PFNA, PFDA, PFUnDA, and PFDoA. These seven substances belong to perfluoroalkyl carboxylic acid (PFCA). From PFHxA (carbon chain length C6) to PFDoA (C12), there is a trend that the BAF value of mango gradually decreases as the carbon chain length increases. However, PFOS belongs to perfluoroalkyl sulfonic acid (PFSA) and does not follow this trend. Similar results were also found in greenhouse and field studies [10]. This phenomenon primarily stems from two mechanistic barriers: (1) membrane permeability limitations—long-chain PFCAs face difficulty crossing cell membranes due to their bulky molecular structures [34], requiring inefficient protein-mediated transport mechanisms that reduce cellular uptake and consequently lower BAF values [35]; (2) vascular transport constraints—the vascular bundle system demonstrates limited capacity for long-chain PFCA translocation, exhibiting both low absorption into vascular tissues and impaired root-to-shoot transport. This likely results from either weak affinity with vascular bundle cells or excessive adsorption in intercellular spaces, collectively reducing aboveground accumulation and BAF values.

### 3.4. Effects of Soil pH and Soil Organic Matter on PFAS Transport in Soil

Correlation analyses revealed significant relationships between soil PFAS concentrations and both pH (4.5–7.5) and organic matter content (10.8–48.2 g/kg) (Table S3). PFHpA, PFOA, PFNA, and PFOS exhibited negative correlations with soil pH, as acidic conditions promote positive soil surface charges that enhance anionic PFAS adsorption [36]. Conversely, alkaline conditions increase negative surface charges, reducing adsorption through electrostatic repulsion. Notably, PFDoA and PFDA exhibited positive correlations with pH (*p* < 0.01 and *p* < 0.05, respectively). Their extended perfluorocarbon chains enhance hydrophobicity, favoring organic matter interactions that dominate over electrostatic effects [37]. Higher pH facilitates organic matter conformational changes that expose hydrophobic binding sites, sustaining adsorption despite increased molecular negative charges [38]. All measured PFAS correlated positively with organic matter, particularly PFNA and PFOS (*p* < 0.05). Organic matter provides adsorption sites through hydrophobic/electrostatic interactions, increasing soil retention while reducing plant bioavailability [39]. Organic matter offers adsorption sites for PFAS and binds to their molecules through mechanisms like hydrophobic and electrostatic interactions, retaining more PFASs in the soil but potentially limiting plant uptake.

### 3.5. Analysis of Possible Sources of PFAS Contamination in Soil

To further investigate the potential sources of PFAS in soil, PCA was employed to identify the sources of the eight target PFAS congeners. As shown in Table S4, more than 82% of the total variance in soil PFAS concentrations could be explained by two factors. Factor 1, characterized by high factor loadings for PFHpA and PFHxA, accounted for 64.8% of the variance in PFAS sources across soil samples. Both PFHpA and PFHxA are short-chain PFAS, predominantly derived from atmospheric deposition [40]. Factor 2 exhibited high loadings for PFDA, PFDoA, PFOA, and PFUnDA, collectively contributing 18.1% to the total variance. The high proportions of PFDA, PFOA, and PFUnDA are mainly attributed to volatile precursors such as fluorotelomer alcohols (FTOHs) and perfluorosulfonamides. These precursors can volatilize from polymer products into the atmosphere, undergo long-range atmospheric circulation, and subsequently form the target PFAS via atmospheric chemical reactions or biodegradation [41]. In contrast, PFDoA is likely generated from the oxidation of FTOHs present in the integrated adhesives of leather and paper products [42].

**Figure 4:**
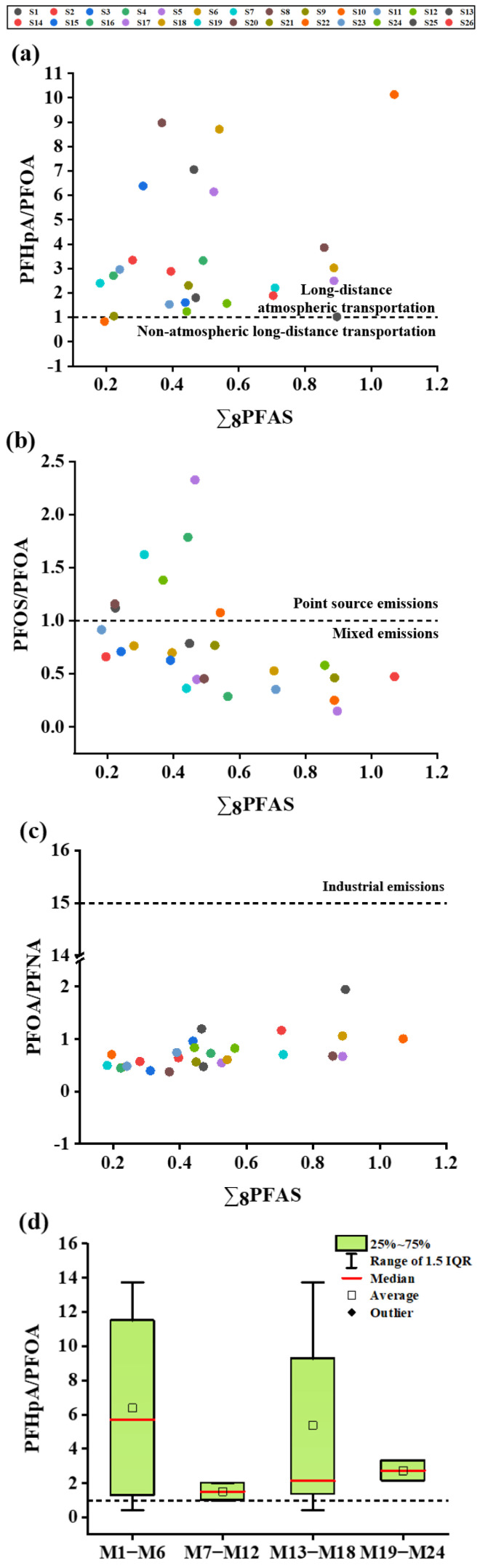
Ratios of PFHpA/PFOA (**a**), PFOS/PFOA (**b**), and PFOA/PFNA (**c**) in soil. PFHpA/PFOA ratio (**d**) in mangoes. The box and whisker chart displays the PFHpA/PFOA ratios of individual mangoes, with M1–M24 representing mango sampling points. The boxes represent the 25th to 75th percentile lines. The solid red line inside the box represents the median, and the black hollow box represents the average value.

In order to identify PFAS sources in soil, we calculated PFHpA/PFOA, PFOS/PFOA, and PFOA/PFNA ratios as indicators of atmospheric long-range transport, point source input, and industrial emissions, respectively [40,42]. As shown in Figure 4a, the ratio of PFHpA/PFOA in all soil samples except S10 was greater than 1, indicating that atmospheric deposition was the main source of PFAS pollution in soil. Figure 4b shows that the PFOS/PFOA ratio of 73.1% of all soil samples was less than 1, indicating that the distribution of PFAS in soil was mainly affected by the mixing of multiple input pathways. The PFOA/PFNA ratio results in Figure 4c showed that the PFAS source in soil may be related to the release of commercial products and consumer products, and industrial emissions have a minimal influence. Given that PFASs are commonly used as additives in waterproof and oil-resistant food packaging materials [43], the present study concomitantly analyzed PFASs in mango bags. The results indicated that these bags were dominated by legacy PFASs. In practical production, farmers directly discard used mango bags on the soil’s surface after mango harvest without timely cleanup, with an average exposure duration of 3–6 months, resulting in long-term direct contact between mango bags and soil. Therefore, mango bags, as a commercial product, represent one of the non-negligible sources of PFASs in soil. Soil PFAS pollution originates from multiple sources, with long-range atmospheric transport being predominant. Due to their volatility, PFASs persist in the atmosphere during production, use, and disposal, undergoing long-distance transport via atmospheric circulation. These compounds interact with airborne particles and ultimately deposit via dry and wet deposition processes.

### 3.6. Analysis of Possible Sources of PFASs in Mango

In this study, the BAF of different PFAS, the ratio of PFHpA/PFOA in mango, and the content of PFAS in mango bags were analyzed to explore the possible sources of PFAS in mango. The bioaccumulation characteristics of PFASs in mango have been described in 3.3. The results showed that mango has a weak ability to enrich PFAS in soil, and PFASs have difficulty entering mangoes in large quantities. The ratio of PFHpA to PFOA in mango is shown in Figure 4d. The results showed that 83% of mango PFHpA/PFOA ratios were greater than 1 in 24 mango samples, indicating that atmospheric deposition is also the main source of PFASs in mangoes. Exhaust gases containing PFASs are transported over long distances through the atmosphere, and dry and wet deposition occurs in mango orchards, allowing these substances to enter the soil and adhere to the surface of mangoes. The ratio of PFHpA to PFOA of the remaining 17% of mango is lower than 1, indicating that PFASs in mango have other pollution sources besides atmospheric deposition, which may come from agricultural inputs. Some fertilizers and pesticides may contain PFASs as impurities or additives [20], which adhere to the surface of the mango during application and are then absorbed by the mango.

**Figure 5:**
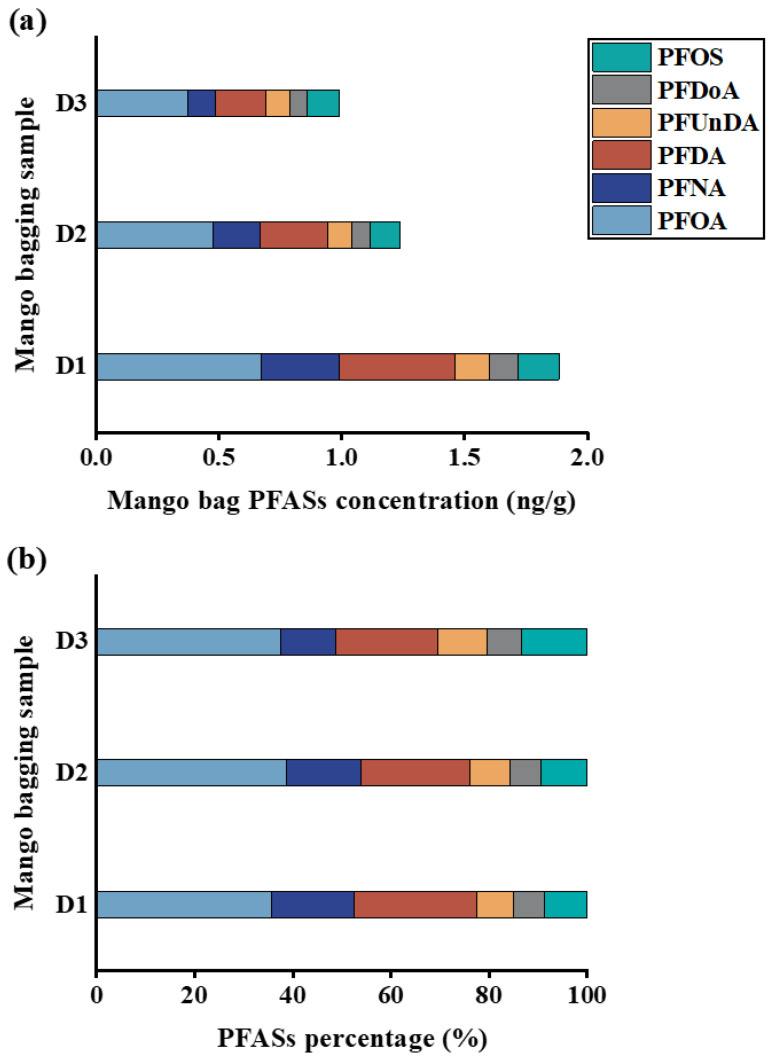
The concentration and proportion of PFASs in mango bags. (**a**) The sum of all PFAS concentrations; (**b**) proportion of PFASs.

In addition, this study analyzed the PFAS contents in mango bags. As shown in Figure 5a, six PFAS compounds, including PFOA, PFNA, PFDA, PFUnDA, PFDoA, and PFOS, were detected in mango bags, with the total concentration ranging from 0.99 to 1.88 ng/g and the average concentration being 1.37 ng/g. The detection rates of PFOA, PFDA, and PFNA were 100%, and the detected concentrations were 0.37–0.67 ng/g, 0.21–0.47 ng/g, and 0.11–0.31 ng/g, respectively. In addition to legacy PFASs, the results in Figure 5b show that short-chain PFASs (PFHpA and PFHxA) were not detected. The above results indicate that mango bags are one of the possible sources of legacy PFASs in mangos besides atmospheric deposition. 

### 3.7. Soil Ecological Risk Assessment

As there are few reports on the standard ecological risk limits of PFAS in soil, this study refers to the toxicity data of target PFAS to aquatic organisms and their soil–water partition coefficient in existing studies. The environmental no-effect concentrations (PNEC) of the target compounds PFHxA, PFHpA, PFOA, PFNA, PFDA, PFUnDA, PFDoA, and PFOS were derived using the evaluation factors in soil. They were 19.14, 91.18, 6.34, 4.96, 2.11, 4.52, 8.55, and 1.09 mg/kg, respectively [44]. The risk quotient (RQ) was obtained by dividing the concentration of PFAS detected in soil by the derived PNEC to evaluate the ecological risk of PFAS. As shown in Table 2, the RQ values of PFAS in soil are much lower than 0.01, indicating a relatively low ecological risk. Since the main source of PFAS pollution in soil is atmospheric deposition, and it is affected by multi-channel mixed discharge, the ecological risk of PFAS in the soil should be continuously monitored. In addition, other possible sources of pollution, such as pesticides and fertilizers, should be further explored for their negative effects.

### 3.8. Human Health Risk Assessment

PFASs in mango enter the human body through the food chain and may cause adverse effects on human health [31]. Therefore, it is necessary to assess the risk level of human exposure to PFAS through the consumption of mango. As shown in Table 2, the EDI of average concentrations of PFAS in mangoes ranged from 0.0001 to 0.0035 ng/kg/d, with the highest EDI value for PFHxA being 0.0095 ng/kg/d. This was followed by PFHpA at 0.0035 ng/kg/d and PFOS at 0.0024 ng/kg/d. The EDI of other legacy PFCAs, including PFOA, PFNA, PFDA, and PFUnDA, is much lower than that of PFHxA and PFHpA, suggesting that PFHxA and PFHpA are more likely to enter the human body through mango consumption than legacy PFCAs. The maximum RQ values of PFHxA, PFOA, and PFOS were 0.00003, 0.00051, and 0.0012, respectively, all of which were much less than 1, indicating a low risk of human exposure to PFAS through mango consumption. Compared to other types of food, the EDI of each target PFAS in mangoes was several orders of magnitude lower than the EDI of each target PFAS in various foods such as grains, vegetables, eggs, meat, and fish [12]. This discrepancy is primarily attributed to the absence of local pollution sources near the mango cultivation area, which minimizes direct exposure to industrial discharges. In contrast, previous studies conducted near PFAS-contaminated hotspots (e.g., fluorochemical manufacturing facilities) reported significantly elevated bioaccumulation levels in food matrices due to proximity to point-source emissions. Therefore, the results of this study well reflect the EDI value of PFAS in mangoes planted in normal soil, indicating that the potential risk of PFAS in mangoes in this region to humans is extremely low at present. However, it is important to note that although the current PFAS exposure risk from mangoes in this region is extremely low, PFASs are characterized by high persistence and bioaccumulation potential, with low metabolic excretion efficiency in the human body. Long-term ingestion of multiple daily foods containing low doses of PFASs, including mangoes (such as grains, vegetables, and dairy products), may lead to the gradual accumulation of these pollutants in the human body, resulting in a combined exposure effect. As the accumulation increases, even if the exposure dose from a single food is at a low-risk level, the cumulative effect may still exceed the human safe tolerance threshold, thereby increasing the potential health risks such as metabolic disorders and endocrine disruption [2,3]. Furthermore, the long-term toxicological effects of short-chain PFASs remain to be further investigated. In the future, continuous monitoring of the pollution sources and risk dynamics of PFASs in mangoes is still necessary. The risk assessment paradigm established in this study also provides a practical reference for the safety supervision of persistent pollutants in similar tropical agricultural products and holds significant practical value for improving the food safety system.

## 4. Conclusions

In this study, soil and mango samples were collected from mango bases in Nanfan District to investigate the main types, accumulation levels, pollution sources, and bioaccumulation characteristics of PFASs in the two matrices. Further, the ecological risks and human health risks associated with these substances were evaluated. In the present study, the contamination levels of short-chain PFASs in the soil were higher than those of legacy PFASs. The content of PFASs in mango soil was much lower than that of contaminated soil. PFAS pollution in soil was mainly affected by multiple emission sources, and the main source was long-distance atmospheric transport. The concentration of PFASs in mango was relatively low, with PFHxA and PFHpA as the main compounds. The accumulation ability of PFASs in mango was weak, and it was difficult for PFASs to enter mangos in large quantities from the soil. The main source of PFASs in mangoes was similar to that in soil, mainly atmospheric deposition. The results of this study have, for the first time, systematically clarified the pollution characteristics, migration patterns, and source mechanisms of PFASs in soil–mango systems under tropical climatic conditions. This breaks the limitations of previous studies on agricultural PFAS pollution, which have focused primarily on temperate crops and industrial contaminated areas, thereby enriching the global PFAS pollution database with cases of tropical agricultural ecosystems. Additionally, this study confirmed the dominant status of short-chain PFASs in tropical fruits, providing critical references for the environmental impact assessment following the implementation of global PFAS replacement policies. It has also identified mango bags as a novel pollution source, offering practical targets for pollution prevention and control in tropical agricultural production. Although the current results of ecological risk assessment for soil PFASs and human health risk assessment associated with mango consumption indicate low risks, continuous monitoring of risk levels remains necessary as the usage of PFAS evolves and their accumulation persists. Furthermore, a limitation of this study lies in its single-time sampling, which may fail to capture the seasonal variations in PFAS concentrations. Future research should conduct long-term monitoring targeting different growth stages of mangoes to explore the migration and transformation patterns of PFASs during these stages. It is also essential to expand the research scope to other tropical fruits such as bananas and pineapples, aiming to reveal the commonalities and specificities of PFAS accumulation in tropical crops. Additionally, in-depth analysis of the interface behaviors and toxicological effects of short-chain PFASs in the tropical soil–plant system is required to provide more comprehensive scientific support for formulating targeted pollution control standards and agricultural production regulations.

## Figures and Tables

**Table 1:** PFAS concentration ranges and predominant components in crops grown in soils with different backgrounds.

Sample Types	Location	ΣPFAS Concentration Range	Dominant PFAS	Reference
Mangoes	Hainan Nanfan District, China (Normal agricultural soil)	0.0019–0.0201 ng/g (ww)	PFHxA, PFHpA	This study
Fruits (strawberries, apples, pears)	Netherlands (Normal agricultural soil)	<0.03 ng/g	No dominant congeners	[25]
Vegetables (Cucumbers, cauliflowers)	Netherlands (Normal agricultural soil)	0.007 ng/g (mean)	PFUnDA, PFOS, PFDA	[26]
Maize kernels	Farmland near the mega fluorine chemical industrial park in China	1.36–58.83 ng/g	PFBA	[10]
Cabbages	Farmland near the mega fluorine chemical industrial park in China	11.88–115.14 ng/g	PFBA	[27]

**Table 2:** Assessment of soil ecological risks and human health risks.

Analytes	Soil Ecological Risk		Human Health Risk			
	RQ (×10^−6^)		EDI (×10^−3^)		RQ (×10^−4^)	
	Max	Min	Max	Min	Max	Min
PFHxA	21.82	0.98	9.47	1.30	0.30	0.04
PFHpA	4.44	0.20	3.50	0.17	—	—
PFOA	20.22	1.77	1.52	0.03	5.07	0.11
PFNA	30.81	4.02	0.15	0.02	—	—
PFDA	148.75	6.41	0.58	0.58	—	—
PFUnDA	26.37	4.70	0.49	0.01	—	—
PFDoA	2.81	0.24	—	—	—	—
PFOS	78.77	7.30	2.35	0.57	11.77	2.84

## Data Availability

The original contributions presented in this study are included in this article and Supplementary Materials. Further inquiries can be directed to the corresponding authors.

## Supplementary Materials

The following supporting information can be downloaded at https://www.mdpi.com/article/10.3390/foods15010058/s1. Figure S1: Sampling sites in Nanfan District (NFD) in Hainan, China; Table S1: Mass spectrometry multi-reaction monitoring conditions; Table S2: Analyses of target PFASs measured in the study with QA/QC information; Table S3: Pearson correlation analysis of PFASs concentration in mango soil; Table S4: Loading factors of PFASs in soil of mango base.

## References

[1] Barhoumi B., Sander S.G., Tolosa I. (2022). A review on per-and polyfluorinated alkyl substances (PFASs) in microplastic and food-contact materials. Environ. Res..

[2] Martínez-Esquivel A., Trujillo-Silva D.J., Cilia-López V.G. (2022). Impact of environmental pollution on the obesogenic environment. Nutr. Rev..

[3] Panieri E., Baralic K., Djukic-Cosic D., Djordjevic A.B., Saso L. (2022). PFAS molecules: A major concern for the human health and the environment. Toxics.

[4] Stahl T., Riebe R.A., Falk S., Failing K., Brunn H. (2013). Long-term lysimeter experiment to investigate the leaching of perfluoroalkyl substances (PFASs) and the carry-over from soil to plants: Results of a pilot study. J. Agric. Food Chem..

[5] Xu P., Nian M., Xiang J., Zhang X.H., Cheng P., Xu D.D., Chen Y., Wang X.F., Chen Z.J., Lou X.M. (2025). Emerging PFAS Exposure Is More Potent in Altering Childhood Lipid Levels Mediated by Mitochondrial DNA Copy Number. Environ. Sci. Technol..

[6] Cui Y., Wu A.T., Liu H., Zhong Y.Y., Yi K.F. (2025). The effect and potential mechanisms of per-and polyfluoroalkyl substances (PFAS) exposure on kidney stone risk. Ecotoxicol. Environ. Saf..

[7] Bassler J., Ducatman A., Elliott M., Wen S.J., Wahlang B., Barnett J., Cave M.C. (2019). Environmental perfluoroalkyl acid exposures are associated with liver disease characterized by apoptosis and altered serum adipocytokines. Environ. Pollut..

[8] Mei W.P., Sun H., Song M.K., Jiang L.F., Li Y.T., Lu W.S., Ying G.G., Luo C.L., Zhang G. (2021). Per-and polyfluoroalkyl substances (PFASs) in the soil–plant system: Sorption, root uptake, and translocation. Environ. Int..

[9] Adu O., Ma X.M., Sharma V.K. (2023). Bioavailability, phytotoxicity and plant uptake of per-and polyfluoroalkyl substances (PFAS): A review. J. Hazard. Mater..

[10] Liu Z.Y., Lu Y.L., Song X., Jones K., Sweetman A.J., Johnson A.C., Zhang M., Lu X.T., Su C. (2019). Multiple crop bioaccumulation and human exposure of perfluoroalkyl substances around a mega fluorochemical industrial park, China: Implication for planting optimization and food safety. Environ. Int..

[11] Piva E., Fais P., Ioime P., Forcato M., Viel G., Cecchetto G., Pascali J.P. (2023). Per-and polyfluoroalkyl substances (PFAS) presence in food: Comparison among fresh, frozen and ready-to-eat vegetables. Food Chem..

[12] Yang H., Zhao Y., Chai L.N., Ma F.J., Yu J.L., Xiao K.Q., Gu Q.B. (2024). Bio-accumulation and health risk assessments of per-and polyfluoroalkyl substances in wheat grains. Environ. Pollut..

[13] Godoy A.A., Kummrow F., Pamplin P.A.Z. (2015). Occurrence, ecotoxicological effects and risk assessment of antihypertensive pharmaceutical residues in the aquatic environment-a review. Chemosphere.

[14] Chen X.Y., Li J., Han L., Wu W.P., Chen M.F. (2023). Human health risk-based soil generic assessment criteria of representative perfluoroalkyl acids (PFAAs) under the agricultural land use in typical Chinese regions. Environ. Pollut..

[15] Yang D.W., Han J.J., Hall D.R., Sun J.X., Fu J., Kutarna S., Houck K.A., Lalone C.A., Doering J.A., Ng C.A. (2020). Nontarget screening of per- and polyfluoroalkyl substances binding to human liver fatty acid binding protein. Environ. Sci. Technol..

[16] Ma D.H., Zhong H.F., Lv J.T., Wang Y.W., Jiang G.B. (2022). Levels, distributions, and sources of legacy and novel per-and perfluoroalkyl substances (PFAS) in the topsoil of Tianjin, China. J. Environ. Sci..

[17] Cheng Y., An Q., Qi H.S., Li R., Liu W.P., Gu B.J., Liu K. (2023). Temporal trends of legacy and emerging PFASs from 2011 to 2021 in agricultural soils of eastern China: Impacts of the Stockholm convention. Environ. Sci. Technol..

[18] Li H., Koosaletse-Mswela P. (2023). Occurrence, fate, and remediation of per-and polyfluoroalkyl substances in soils: A review. Curr. Opin. Environ. Sci. Health.

[19] Zhu J.C., Fu Y., Hu H., Zhong Y.S., Ma X., Zhu Y.L., Zhou F., Pan Y.T., Ma Y.X. (2025). Regulation of terrestrial input and ocean processes on the occurrence and transport of traditional and emerging per-and polyfluoroalkyl substances in the inner shelf of the East China Sea. Water Res..

[20] Saliu T.D., Liu M., Habimana E., Fontaine J., Dinh Q.T., Sauvé S. (2024). PFAS profiles in biosolids, composts, and chemical fertilizers intended for agricultural land application in Quebec (Canada). J. Hazard. Mater..

[21] Oliver D.P., Li Y.S., Orr R., Nelson P., Barnes M., McLaughlin M.J., Kookana R.S. (2020). Sorption behaviour of per-and polyfluoroalkyl substances (PFASs) in tropical soils. Environ. Pollut..

[22] Xie S.W., Lu Y.L., Wang T.Y., Liu S.J., Jones K., Sweetman A. (2013). Estimation of PFOS emission from domestic sources in the eastern coastal region of China. Environ. Int..

[23] Liu Z.Y., Liu S., Xiao F., Sweetman A.J., Cui Q.Q., Guo H., Xu J.Y., Luo Z.Y., Wang M.X., Zhong L.L. (2024). Tissue-specific distribution and bioaccumulation of perfluoroalkyl acids, isomers, alternatives, and precursors in citrus trees of contaminated fields: Implication for risk assessment. J. Hazard. Mater..

[24] Chen L.F., Chen Y.B., Zhang Y.Y., Xu S.C. (2022). Spatial patterns of typhoon rainfall and associated flood characteristics over a mountainous watershed of a tropical island. J. Hydrol..

[25] Pancras T., Bentum E., Pagter L., Hoef M., Hoogenboom R., Berendsen B., Leeuwen S.P.J. (2024). Large scale study on PFASs levels in fruits, vegetables and soil from allotments and gardens contaminated by atmospheric deposition from a Dutch fluorochemical production plant. Chemosphere.

[26] Risk Assessment of Exposure to PFAS Through Food and Drinking Water in the Netherlands. https://rivm.openrepository.com/server/api/core/bitstreams/372952eb-a911-41dc-b29f-6249702f989f/content.

[27] Xu C., Song X., Liu Z.Y., Ding X.Y., Chen H., Ding D. (2021). Occurrence, source apportionment, plant bioaccumulation and human exposure of legacy and emerging per-and polyfluoroalkyl substances in soil and plant leaves near a landfill in China. Sci. Total Environ..

[28] Liu N., Li M.Y. (2024). Distinctive adsorption and transport behaviors of short-chain versus long-chain perfluoroalkyl acids in a river sediment. Environ. Sci. Pollut. Res..

[29] Lalonde S., Wipf D., Frommer W.B. (2004). Transport mechanisms for organic forms of carbon and nitrogen between source and sink. Annu. Rev. Plant Biol..

[30] Bresinsky A., Körner C., Kadereit J.W., Neuhaus G., Sonnewald U. (2008). Strasburger, Lehrbuch der Botanik für Hochschulen. 36. Aufl/neu bearb. von Andreas Bresinsky.

[31] Blaine A.C., Rich C.D., Sedlacko E.M., Hyland K.C., Stushnoff C., Dickenson E.R., Higgins C.P. (2014). Perfluoroalkyl acid uptake in lettuce (*Lactuca sativa*) and strawberry (*Fragaria ananassa*) irrigated with reclaimed water. Environ. Sci. Technol..

[32] Lechner M., Knapp H. (2011). Carryover of perfluorooctanoic acid (PFOA) and perfluorooctane sulfonate (PFOS) from soil to plant and distribution to the different plant compartments studied in cultures of carrots (*Daucus carota* ssp. sativus), potatoes (*Solanum tuberosum*), and cucumbers (*Cucumis sativus*). J. Agric. Food Chem..

[33] Stahl T., Heyn J., Thiele H., Hüther J., Failing K., Georgii S., Brunn H. (2009). Carryover of perfluorooctanoic acid (PFOA) and perfluorooctane sulfonate (PFOS) from soil to plants. Arch. Environ. Contam. Toxicol..

[34] Felizeter S., McLachlan M.S., De Voogt P. (2012). Uptake of perfluorinated alkyl acids by hydroponically grown lettuce (*Lactuca sativa*). Environ. Sci. Technol..

[35] Lan Z.H., Zhou M., Yao Y.M., Sun H.M. (2018). Plant uptake and translocation of perfluoroalkyl acids in a wheat–soil system. Environ. Sci. Pollut. Res..

[36] Campos-Pereira H., Kleja D.B., Ahrens L., Enell A., Kikuchi J., Pettersson M., Gustafsson J.P. (2023). Effect of pH, surface charge and soil properties on the solid–solution partitioning of perfluoroalkyl substances (PFASs) in a wide range of temperate soils. Chemosphere.

[37] Nguyen T.M.H., Bräunig J., Thompson K., Thompson J., Kabiri S., Navarro D.A., Kookana R.S., Grimison C., Barnes C.M., Higgins C.P. (2020). Influences of chemical properties, soil properties, and solution pH on soil–water partitioning coefficients of per-and polyfluoroalkyl substances (PFASs). Environ. Sci. Technol..

[38] Campos-Pereira H., Kleja D.B., Sjöstedt C., Ahrens L., Klysubun W., Gustafsson J.P. (2020). The adsorption of per-and polyfluoroalkyl substances (PFASs) onto ferrihydrite is governed by surface charge. Environ. Sci. Technol..

[39] Cai W.W., Navarro D.A., Du J., Ying G.G., Yang B., McLaughlin M.J., Kookana R.S. (2022). Increasing ionic strength and valency of cations enhance sorption through hydrophobic interactions of PFAS with soil surfaces. Sci. Total Environ..

[40] Zhong H.F., Zheng M.G., Liang Y., Wang Y.J., Gao W., Wang Y.W., Jiang G.B. (2021). Legacy and emerging per-and polyfluoroalkyl substances (PFAS) in sediments from the East China Sea and the Yellow Sea: Occurrence, source apportionment and environmental risk assessment. Chemosphere.

[41] Young C.J., Furdui V.I., Franklin J., Koerner R.M., Muir D.C., Mabury S.A. (2007). Perfluorinated acids in arctic snow: New evidence for atmospheric formation. Environ. Sci. Technol..

[42] Simcik M.F., Dorweiler K.J. (2005). Ratio of perfluorochemical concentrations as a tracer of atmospheric deposition to surface waters. Environ. Sci. Technol..

[43] Glenn G., Shogren R., Jin X., Orts W., Hart-Cooper W., Olson L. (2021). Per-and polyfluoroalkyl substances and their alternatives in paper food packaging. Compr. Rev. Food Sci. Food Saf..

[44] Xiang L., Li Y.W., Yu P.F., Feng N.X., Zhao H.M., Li H., Cai Q.Y., Mo C.H., Li Q.X. (2019). Food safety concerns: Crop breeding as a potential strategy to address issues associated with the recently lowered reference doses for perfluorooctanoic acid and perfluorooctane sulfonate. J. Agric. Food Chem..

