# Supplementary material for: Occurrence, Sources, and Risk Assessment of PFAS in Soil–Mango Systems of the Chinese Tropical Nanfan District
Source: Foods. 2025 Dec 24;15(1):58. doi: 10.3390/foods15010058 (PMC12785708; doi:10.3390/foods15010058)
Supplement: Supplementary file 1 [file foods-15-00058-s001.zip › foods-4024779-supplementary.pdf]

Supplementary Information

Occurrence, Sources, and Risk Assessment of PFAS in Soil–Mango Systems of the Chinese Tropical Nanfan District

Zhen Zhang [1,†], Fei Chen [2,†], Rui Yang [3], Saihao Ren [4], Shanying Zhang [5], Xiaowei Pan [4], Hai Tian [1], Thiagarajah Ramilan [6], Yun Duan [1,*] and Bingjun Han [1,*]

[1] Analysis and Test Center, Chinese Academy of Tropical Agricultural Sciences, Key Laboratory of Quality and Safety Control for Subtropical Fruit and Vegetable, Ministry of Agriculture and Rural Affairs, Hainan Provincial Key Laboratory of Quality and Safety for Tropical Fruits and Vegetables, Key Laboratory of Nutritional Quality and Health Benefits of Tropical Agricultural Products of Haikou City, Laboratory of Quality & Safety Risk Assessment for Tropical Products (Haikou), Ministry of Agriculture and Rural Affairs, Haikou 571101, China; 18145123688@163.com (Z.Z.); tianhai666@163.com (H.T.)

[2] School of Food Science and Engineering, Hainan University, Haikou 570228, China; 18299151539@163.com

[3] Hainan Ecological Environmental Monitoring Center, Haikou 572000, China; 21180311019@stu.ouc.edu.cn

[4] Agricultural Products Processing Research Institute, Chinese Academy of Tropical Agricultural Sciences, Zhanjiang 524001, China; rshkyzy@foxmail.com (S.R.); panxiaowei@catas.cn (X.P.)

[5] Sanya Nanfan Research Institute of Hainan University, School of Tropical Agriculture and Forestry (School of Agricultural and Rural Affairs, School of Rural Revitalization), Hainan University, Haikou 570228, China; 17889986721@163.com

[6] School of Agriculture and Environment, Massey University, Palmerston North 4442, New Zealand; t.ramilan@massey.ac.nz

* Correspondence: catas.duanyun@hotmail.com (Y.D.); hanbjun@163.com (B.H.)

† These authors equally contributed to this paper.

Soil organic matter was determined as follows:

Soil organic matter was determined as follows: Air-dried soil samples (passed through a 0.25-mm sieve) were accurately weighed (0.05–0.5 g, precision: 0.0001 g). A 10.00-mL aliquot of 0.4 mol/L potassium dichromate-sulfuric acid solution was added, and the mixture was shaken; a glass funnel was placed on each test tube mouth.Test tubes were placed in a wire cage and immersed in an oil bath preheated to 185–190°C, with the liquid level in tubes below the oil level. After immersion, the oil bath temperature was maintained at 170–180°C. Timing started when the solution boiled; the temperature was controlled to avoid vigorous boiling, and the cage was gently shaken occasionally for uniform heating. After 5 ± 0.5 min, the cage was removed and cooled briefly.Oil residue on test tubes was wiped off. The digested solution and soil residues were transferred to a 250-mL Erlenmeyer flask; the test tube and funnel were rinsed with water, and rinsates were combined to adjust the total volume to 50–60 mL. Three drops of phenanthroline indicator were added, and the remaining $K_2Cr_2O_7$ was titrated with standard ferrous sulfate solution, with the endpoint indicated by a color change from orange-yellow to blue-green to reddish-brown. Soil organic matter (O.M, g/kg) was calculated as:

$$O.M = \frac{c \times (V0 - V) \times 0.003 \times 1.724 \times 1.10}{m} \times 1000$$

Where V_0 and V are the volumes (mL) of ferrous sulfate standard solution consumed in blank and sample titrations, respectively; c is the concentration of ferrous sulfate (mol/L); 0.003 is the millimolar mass of 1/4 C (g); 1.724 converts organic C to organic matter; 1.10 is the oxidation correction factor; and m is the mass of oven-dried soil (g).

Soil pH was determined as follows:

Air-dried soil samples (passed through a 2-mm sieve) were accurately weighed (10.0 ± 0.1 g) into 50-mL tall-form beakers or other suitable containers, followed by addition of 25 mL water (or potassium

chloride/calcium chloride solution). Containers were sealed, vigorously shaken or stirred for 5 min using a shaker or stirrer, then allowed to stand for 1–3 h. pH was measured using a pH meter.

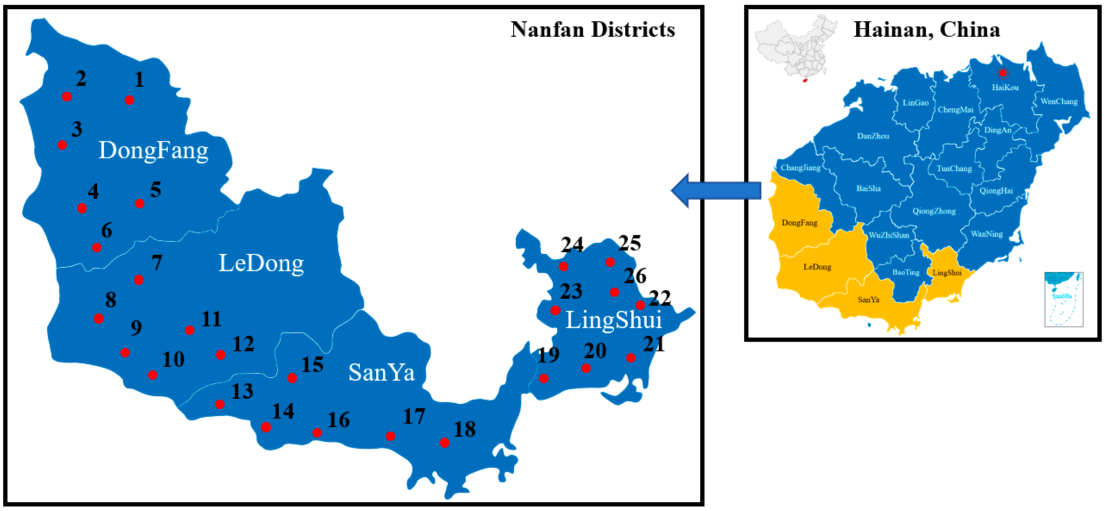

Figure S1. Sampling sites in Nanfan District (NFD) in Hainan, China.

Table S1. Mass spectrometry multi-reaction monitoring conditions.

Compound	Formula	Precursor Ion [M-H]-(amu)	Precursor Ion (amu)	DP (volts)	CE (volts)
PFHxA	$[C_6HF_{11}O_2]$	313.0	269.0	-25	-14
PFHxA	$[C_6HF_{11}O_2]$	313.0	118.9	-25	-26
PFHpA	$[C_7HF_{13}O_2]$	363.0	319.0	-45	-14
PFHpA	$[C_7HF_{13}O_2]$	363.0	168.9	-45	-28
PFOA	$[C_8HF_{15}O_2]$	413.0	368.9	-25	-16
PFOA	$[C_8HF_{15}O_2]$	413.0	168.9	-25	-27
PFNA	$[C_9HF_{17}O_2]$	463.0	419.0	-30	-17
PFNA	$[C_9HF_{17}O_2]$	463.0	219.0	-30	-25
PFDA	$[C_{10}HF_{19}O_2]$	513.0	469.0	-30	-19
PFDA	$[C_{10}HF_{19}O_2]$	513.0	218.9	-30	-26
PFUnDA	$[C_{11}HF_{21}O_2]$	563.0	519.0	-45	-19
PFUnDA	$[C_{11}HF_{21}O_2]$	563.0	268.9	-45	-28

PFDoDA	$[C_{12}HF_{23}O_2]$	613.0	569.0	-25	-21
PFDoDA	$[C_{12}HF_{23}O_2]$	613.0	168.9	-25	-34
PFHxS	$[C_6HF_{13}O_3S]$	399.0	79.9	-65	-82
PFHxS	$[C_6HF_{13}O_3S]$	399.0	98.9	-65	-63
PFOS	$[C_8HF_{17}SO_3]$	499.0	79.9	-45	-98
PFOS	$[C_8HF_{17}SO_3]$	499.0	98.9	-45	-85
13C4 PFBA	$[13C_4HF_7O_2]$	217.0	172.0	-25	-14
13C4 PFOA	$[l3C4C_4HF_{15}O_2]$	417.0	372.0	-25	-14
13C4 PFOS	$[13C4C_4F\ _{17}SO_3Na]$	503.0	80.0	-45	-100
13C4 PFOS	$[13C4C_4F\ _{17}SO_3Na]$	503.0	99.0	-45	-95
13C2 PFDA	$[13C2C_8HF_{19}O_2]$	515.0	470.0	-30	-17
13C2 PFDoDA	$[13C2C_{10}HF_{23}O_2]$	615.0	570.0	-25	-18
13C2 PFHxA	$[13C2C_4HF_{11}O_2]$	315.0	270.0	-25	-15
18O2 PFHxS	$[18O2C_6HF_{13}OSNa]$	403.0	84.0	-65	-80
18O2 PFHxS	$[18O2C_6HF_{13}OSNa]$	403.0	103.0	-65	-75
13C5 PFNA	$[13C5C_4HF_{17}O_2]$	468.0	423.0	-30	-18
13C5 PFNA	$[13C5C_4HF_{17}O_2]$	468.0	219.0	-30	-25
13C2 PFUnDA	$[13C2C_9HF_{21}O_2]$	565.0	520.0	-45	-20
13C2 PFUnDA	$[13C2C_9HF_{21}O_2]$	565.0	270.0	-45	-28

Table S2. Analyses of target PFASs measured in the study with QA/QC information.

Analytes	Instrument（pg）		Soil (ng/g dw)				Mango (ng/g ww)			
	LOD	LOQ	LOD×10^{-3}	LOQ×10^{-3}	MSR	DF	LOD×10^{-3}	LOQ×10^{-3}	MSR	DF
**PFCAs**										
PFHxA	0.104	0.415	0.415	1.66	113±2.63%	92%	0.415	1.66	104±3.61%	50%
PFHpA	0.207	0.828	0.828	3.31	99.3±2.97%	100%	0.828	3.31	89.8±1.38%	91.7%
PFOA	0.168	0.672	0.672	2.69	108±4.76%	100%	0.672	2.69	108±5.18%	54.2%
PFNA	0.0318	0.127	0.127	0.509	94.5±2.75%	100%	0.127	0.509	95.7±2.97%	16.7%
PFDA	0.0666	0.266	0.266	1.07	86.6±0.78%	100%	0.266	1.07	107±0.66%	4.2%
PFUnDA	0.185	0.742	0.742	2.97	112±4.39%	100%	0.742	2.97	96.8±3.15%	16.7%
PFDoA	0.256	1.02	1.02	4.10	97.6±3.32%	100%	1.02	4.10	97.6±1.65%	0%
**PFSAs**										
PFHxS	0.0162	0.0648	0.0648	0.259	82.9±1.87%	0%	0.0648	0.259	89.9±1.54%	0%
PFOS	0.0582	0.233	0.233	0.931	98.7±4.87%	100%	0.233	0.931	97.5±6.74%	54.2%

Note: LOD, the limit of detection; LOQ, the limit of quantification; MSR, matrix spike recoveries; DF, detection frequencies; a, mean±standard error.

Table S3. Pearson correlation analysis of PFASs concentration in mango soil.

	PFHxA	PFHpA	PFOA	PFNA	PFDA	PFUnDA	PFDoA	PFOS	pH	SOM
PFHxA	1									
PFHpA	.731**	1								
PFOA	0.197	.567**	1							
PFNA	0.062	.535**	.807**	1						
PFDA	0.161	0.078	.587**	0.189	1					
PFUnDA	0.045	0.186	.483*	.593**	.539**	1				
PFDoA	0.075	-0.070	0.292	0.148	.738**	.786**	1			
PFOS	0.061	0.228	0.291	0.327	-0.029	0.099	-0.048	1		
pH	0.304	-0.085	-0.085	-0.273	.422*	0.361	.620**	-0.336	1	
SOM	-0.068	0.200	0.177	.467*	-0.243	0.249	-0.132	.544**	-0.368	1

Note: * represents significance level 0.05 (two-tailed test); ** represents significance level 0.01 (two-tailed test); SOM stands for soil organic matter.

Table S4. Loading factors of PFASs in soil of mango base.

	Factor 1	Factor 2
PFHpA	0.971	-0.067
PFHxA	0.860	-0.111
PFNA	0.457	0.325
PFOS	0.199	0.013
PFDA	0.188	0.948
PFDoA	0.036	0.739
PFOA	0.545	0.634
PFUnDA	0.212	0.624

Note: The suitability of the dataset for principal component analysis (PCA) was assessed via the Kaiser-Meyer-Olkin (KMO) measure of sampling adequacy and Bartlett's test of sphericity prior to conducting the analysis. The KMO value was 0.632, which exceeds the widely accepted threshold of 0.6 (indicative of acceptable sampling adequacy for factor-based methods). Bartlett's test of sphericity yielded a chi-square statistic (χ^2) of 142.710 (degrees of freedom [df] = 28, $p < 0.001$). This significant outcome rejects the null hypothesis (i.e., the variable correlation matrix is an identity matrix), confirming adequate inter-variable correlations to support the application of PCA for PFAS source apportionment.
